# Use and perceptions of Artificial Intelligence among adolescents in Bradford, England: Evidence from Born in Bradford: Age of Wonder

**DOI:** 10.12688/wellcomeopenres.27280.1

**Published:** 2026-08-17

**Authors:** Valerie Yap, Amy Orben, Amira Skeggs, John Pickavance, Megan Wood

**Affiliations:** 1 Medical Research Council Cognition and Brain Sciences Unit, University of Cambridge, Cambridge, England, UK; 2 Bradford Institute for Health Research, Bradford Teaching Hospitals NHS Foundation Trust, Bradford, England, UK

**Keywords:** Generative AI; Artificial Intelligence; Adolescents

## Abstract

Generative artificial intelligence (GenAI) tools are increasingly used by adolescents in academic contexts, yet population-level evidence from community samples remains limited, particularly among under-represented populations. We described quantitative data collected in the 2024/25 round of Born in Bradford: Age of Wonder, an ongoing longitudinal study of secondary school pupils in Bradford, UK. Participants in Years 8–10 (aged 12–15) completed self-report surveys on GenAI use frequency across nine purposes, trust in and verification of GenAI outputs, and perceived usefulness for four school subject categories. Responses were summarised by year group and sex. Of 1,443 pupils, 73.5% reported using GenAI for any purpose at least once or twice per year, with 25.5% reporting daily use. School work was the most common purpose (36.6% at least weekly), followed by checking work (32.0%) and getting information (25.3%). Daily use was higher among males than females (28.3% vs 23.2%) and among Year 8 students than Year 9 and Year 10 (34.9% vs 23.8% and 24.1%). 39.4% reported not trusting GenAI outputs at all, while 47.5% reported never verifying them. Perceived usefulness was generally low across all academic subject types, with GenAI use for creative subjects rated least useful (41.1% reported ‘not at all useful’) and problem-solving subjects (e.g., maths) most useful (21.1% rating ‘very useful’). GenAI use is common among adolescents in Bradford, concentrated in academic contexts, with notable variation by year group and sex. The combination of low trust and low verification across the sample highlights a potential gap in GenAI literacy. These findings offer a descriptive baseline from sample of adolescents of predominantly Pakistani (British) ethnicity that are among the highest quintile for deprivation, who are largely absent from existing research. They also provide a foundation for future investigation of how GenAI use relates to educational engagement and wider adolescent outcomes.

## Introduction

Generative artificial intelligence (GenAI) use among children and young people has grown rapidly, driven by the availability of large language model-based tools that are free, accessible, and increasingly embedded in platforms young people already use. ^1^ These tools carry both potential benefits, including on-demand learning support and accessible information, and potential risks, including displacement of human connection, exposure to inaccurate content, and development of critical literacy skills that remain formative in adolescence. ^2^ ^,^ ^3^ Concerns about these risks have already shaped education and safeguarding policy, with the Department for Education issuing its first guidance on the safe use of GenAI in schools and statutory safeguarding guidance addressing GenAI for the first time. ^4^ ^,^ ^5^

These concerns may be especially pronounced for adolescents, who are often among the earliest and most active adopters of emerging digital technologies but remain in a developmental period where cognitive control, critical literacy, and help-seeking behaviours are still taking shape, potentially heightening susceptibility to unintended harms and disruption to learning. ^6^ ^,^ ^7^ Emerging evidence suggests that frequent GenAI use among young people may be associated with reduced independent thinking and delegating effortful practice to GenAI rather than working through tasks independently, with risks including growing reliance on GenAI, poor judgment of their own abilities, and loss of ownership over their work. ^8^ ^–^ ^10^ However, findings remain preliminary and largely drawn from higher education or adult samples and cannot support inference to adolescent populations without representative youth data.

Born in Bradford’s Age of Wonder study provides a unique opportunity to examine adolescent GenAI use within a large, diverse, and population-based cohort of young people living in an urban UK setting, including communities that are systematically underrepresented in existing research on digital technologies. ^11^ The study was conducted with secondary school students across Bradford and combines self-report survey data with school-reported demographic information. This paper draws on these data to describe patterns of GenAI use – including frequency, purpose, trust, and perceived usefulness across school subjects – and explores how these patterns vary across age and sex.

## Methods

We analysed cross-sectional survey data collected in the 2024/25 academic year from participants in Years 8–10 (ages 12–15) across three secondary schools in Bradford. These data were collected as part of a pilot for the latest questionnaire (Age of Wonder Young People’s survey) and included a subset of three schools from the 24 schools that participated in that academic year. Participating schools provided administrative demographic data including year group, sex, ethnicity, free school meals status, and special educational needs status. Where ethnicity was missing from school records, we used data from the self-report survey. Ethnicity was coded to match the Office for National Statistics census group classification. ^12^ Deprivation was defined using the Index of Multiple Deprivation 2019 for the participant’s home address. ^13^

GenAI use was measured across nine purposes. Participants were asked how frequently they used GenAI for each of the following: getting information; checking their work; generating new ideas; creating images, sounds, or music; writing code; getting advice; schoolwork; and for social reasons. Response options were: ‘Daily’; ‘Once or twice per week’; ‘Once or twice per month’; ‘Once or twice per year’; and ‘Never’. We also derived ‘any purpose’ from their highest frequency response option among all eight options.

Trust and verification of GenAI outputs were each measured with a single item. Participants were asked to rate the extent to which they trusted the answers GenAI provides, with response options: ‘Entirely’; ‘Somewhat’; ‘Undecided’; ‘Not really’; and ‘Not at all’. Participants were also asked how often they verified AI-provided answers, with response options: ‘Almost always’; ‘Sometimes’; ‘Every once in a while’; ‘Rarely’; and ‘Never’.

Perceived usefulness of GenAI for school subjects was measured across four domains: writing subjects (e.g., English, languages); problem-solving subjects (e.g., maths); knowledge-based subjects (e.g., geography, science); and creative subjects (e.g., art). Participants rated usefulness on a six-point scale from 1 (‘Not at all useful’) to 6 (‘Very useful’).

The full Age of Wonder 2024/25 Young People’s survey is available in Extended Data. Frequencies are reported as counts and percentages. Confidence intervals (95%) for all estimates are presented in Supplementary Tables. Extended data (including analytical code and aggregate-level data) are available from OSF. ^14^

## Results

The Age of Wonder 2024/25 questionnaire was completed by 1,443 participants across three secondary schools in Bradford, corresponding to a response rate of 68.9% of students at the participating schools. Participant characteristics are summarised in Table 1, compared to all students in Bradford local authority and nationally. The majority of the sample were Asian or Asian British (83.3%), followed by White (4.0%), Black or Black British (3.0%), and mixed or other ethnicities (9.8%). 71.6% of participants were of Pakistani heritage, compared to 4.6% of pupils nationally. 87.7% lived in the most deprived quintile of the English Index of Multiple Deprivation 2019, compared to 23.7% nationally. 37.3% were eligible for free school meals, compared to 25.8% nationally. 8.7% had Special Education Needs (SEN) provision recorded, compared to 18.1% of pupils in state-funded secondary schools nationally, though SEN data were missing for 23.4% of the sample and this figure should be interpreted with caution.

**Table 1.** Sample characteristics.

Variable	Level	N (%)	All secondary school pupils in Bradford local authority (Y8–10), 2024/25	All secondary school pupils in England, 2024/25 (for comparison, %)
Total		1443 (100)	-	-
Academic year group	Year 8	432 (29.9)	33.1	-
	Year 9	521 (36.1)	33.3	-
	Year 10	490 (34.0)	33.6	-
Sex ^a^	Male	635 (44.0)	49.9	50.3
	Female	808 (56.0)	50.1	49.7
Ethnicity ^b^	Asian and Asian British	1202 (83.3)	45.2	13.8
	* Pakistani*	*1033 (71.6)*	*37.3*	*4.6*
	*Other Asian*	*169 (11.7)*	*6.9*	*9.2*
	White	57 (4.0)	43.3	67.7
	*White British*	*18 (1.2)*	*37.7*	*60.2*
	*Other White*	*39 (2.7)*	*5.6*	*7.5*
	Black and Black British	43 (3.0)	3.4	7.1
	Mixed ethnicities	58 (4.0)	4.0	6.9
	Other	40 (2.8)	2.1	2.5
	Missing/unknown	43 (3.0)	0.2	2.0
Free school meals ^c^		538 (37.3)	33.7	25.8
IMD quintile of home address ^d^	1 (most deprived)	1266 (87.7)	53.6	23.7
	2	161 (11.2)	16.5	20.0
	3	13 (0.9)	16.1	18.5
	4	-	7.9	18.2
	5 (least deprived)	-	5.9	19.7
SEN provision ^e^	Yes	126 (8.7)	18.1	16.5
	No	979 (67.8)	81.9	83.5
	Missing	338 (23.4)	-	-

Data for the comparison columns are from the Department for Education 2023/24 school census for state-funded secondary schools, accessed via https://explore-education-statistics.service.gov.uk/.

^a^ school years 8–10.

^b^ school years 7–11.

^c^ school years 8–10.

^d^ calculated from population of 12–15 year-olds, based on mid-2022 population estimates for of Lower Level Super Output Areas in England ( https://www.ons.gov.uk/peoplepopulationandcommunity/populationandmigration/populationestimates/datasets/lowersuperoutputareamidyearpopulationestimates) and IMD 2019 quintiles of LSOAs ( https://www.gov.uk/government/statistics/english-indices-of-deprivation-2019).

^e^ Education, Health and Care (EHC) plans or SEN support for all pupils attending state-funded secondary school in England. SEN: Special Educational Needs.

Around three-quarters of participants (73.5%) reported using GenAI for any purpose at least once or twice a year, with 25.5% reporting daily use ( Table 2, Figure 1). Use varied considerably by purpose **(Supplementary Figures 1–8).** ^14^ School work was the most common application, with 36.6% using GenAI for this at least weekly (59.7% at least once or twice a year). Checking work (32.0% at least weekly) and getting information (25.3% at least weekly) were the next most frequent purposes. Generating new ideas was used at least weekly by 19.6% of participants and getting advice by 12.5%. The least common purposes were writing code; social reasons; and creating images, sounds, or music, with 84.7%, 78.8%, and 73.7% of participants respectively reporting never using GenAI for these purposes.

**Table 2.** Frequency of GenAI use by purpose, sex, and year group. *I use GenAI for* …

Group	Level	Any purpose					Getting information					Checking my work				
		Daily	Once or twice per week	Once or twice per month	Once or twice per year	Never	Daily	Once or twice per week	Once or twice per month	Once or twice per year	Never	Daily	Once or twice per week	Once or twice per month	Once or twice per year	Never
**Total**		**25.5**	**27.5**	**12.6**	**7.9**	**26.5**	**8.7**	**16.6**	**18.6**	**8.0**	**48.1**	**10.9**	**21.1**	**14.1**	**7.4**	**46.5**
Year Group	8	34.9	25.6	15.1	2.3	22.1	9.6	19.3	16.9	8.4	45.8	16.0	24.7	9.9	11.1	38.3
	9	23.8	29.4	10.2	10.2	26.4	9.5	15.9	18.7	7.5	48.4	10.7	19.0	14.2	7.1	49.0
	10	24.1	26.1	14.3	7.3	28.2	7.5	16.3	19.2	8.4	48.5	9.4	22.2	15.4	6.4	46.6
Sex	Female	23.2	26.9	12.8	8.3	28.7	6.1	15.9	17.8	8.1	52.1	8.7	21.6	17.1	7.1	45.5
	Male	28.3	28.3	12.3	7.4	23.8	11.7	17.4	19.6	7.9	43.4	13.6	20.5	10.5	7.8	47.7

Group	Level	Generating new ideas					Creating images, sounds, or music					Writing code				
		Daily	Once or twice per week	Once or twice per month	Once or twice per year	Never	Daily	Once or twice per week	Once or twice per month	Once or twice per year	Never	Daily	Once or twice per week	Once or twice per month	Once or twice per year	Never
**Total**		**8.3**	**11.3**	**15.0**	**8.0**	**57.4**	**4.2**	**8.1**	**7.7**	**6.3**	**73.7**	**2.7**	**3.8**	**5.4**	**3.4**	**84.7**
Year Group	8	10.3	10.3	11.5	9.0	59.0	8.8	8.8	2.5	11.2	68.8	3.8	1.3	10.3	5.1	79.5
	9	8.8	10.8	17.1	6.4	57.0	4.0	9.1	9.9	7.9	69.2	3.7	5.3	4.5	3.7	82.9
	10	7.2	12.2	13.9	9.3	57.4	2.9	6.7	7.1	2.9	80.3	1.3	3.0	4.7	2.6	88.5
Sex	Female	6.8	10.7	14.3	8.1	59.9	3.9	6.1	7.1	5.5	77.5	1.0	3.0	5.0	2.0	89.0
	Male	10.0	12.0	15.8	7.7	54.4	4.6	10.4	8.5	7.3	69.2	4.7	4.7	5.9	5.1	79.7

Group	Level	Getting advice					School work					Social reasons				
		Daily	Once or twice per week	Once or twice per month	Once or twice per year	Never	Daily	Once or twice per week	Once or twice per month	Once or twice per year	Never	Daily	Once or twice per week	Once or twice per month	Once or twice per year	Never
**Total**		**4.3**	**8.2**	**9.4**	**6.8**	**71.4**	**13.7**	**22.9**	**14.1**	**9.0**	**40.3**	**5.0**	**5.4**	**5.9**	**4.9**	**78.8**
Year Group	8	4.9	12.3	9.9	8.6	64.2	20.7	22.0	8.5	9.8	39.0	3.8	6.3	6.3	5.1	78.5
	9	5.7	6.1	10.2	8.6	69.4	13.1	23.5	12.0	10.4	41.0	6.1	4.5	6.6	4.9	77.9
	10	2.5	8.9	8.5	4.2	75.8	11.9	22.6	18.3	7.2	40.0	4.3	6.0	5.2	4.7	79.8
Sex	Female	4.6	7.5	9.1	6.8	72.0	14.4	23.7	14.4	8.3	39.1	4.6	6.6	4.3	5.0	79.5
	Male	3.9	9.0	9.8	6.7	70.6	12.9	21.9	13.7	9.8	41.8	5.5	3.9	7.9	4.7	78.0

**Figure 1.**
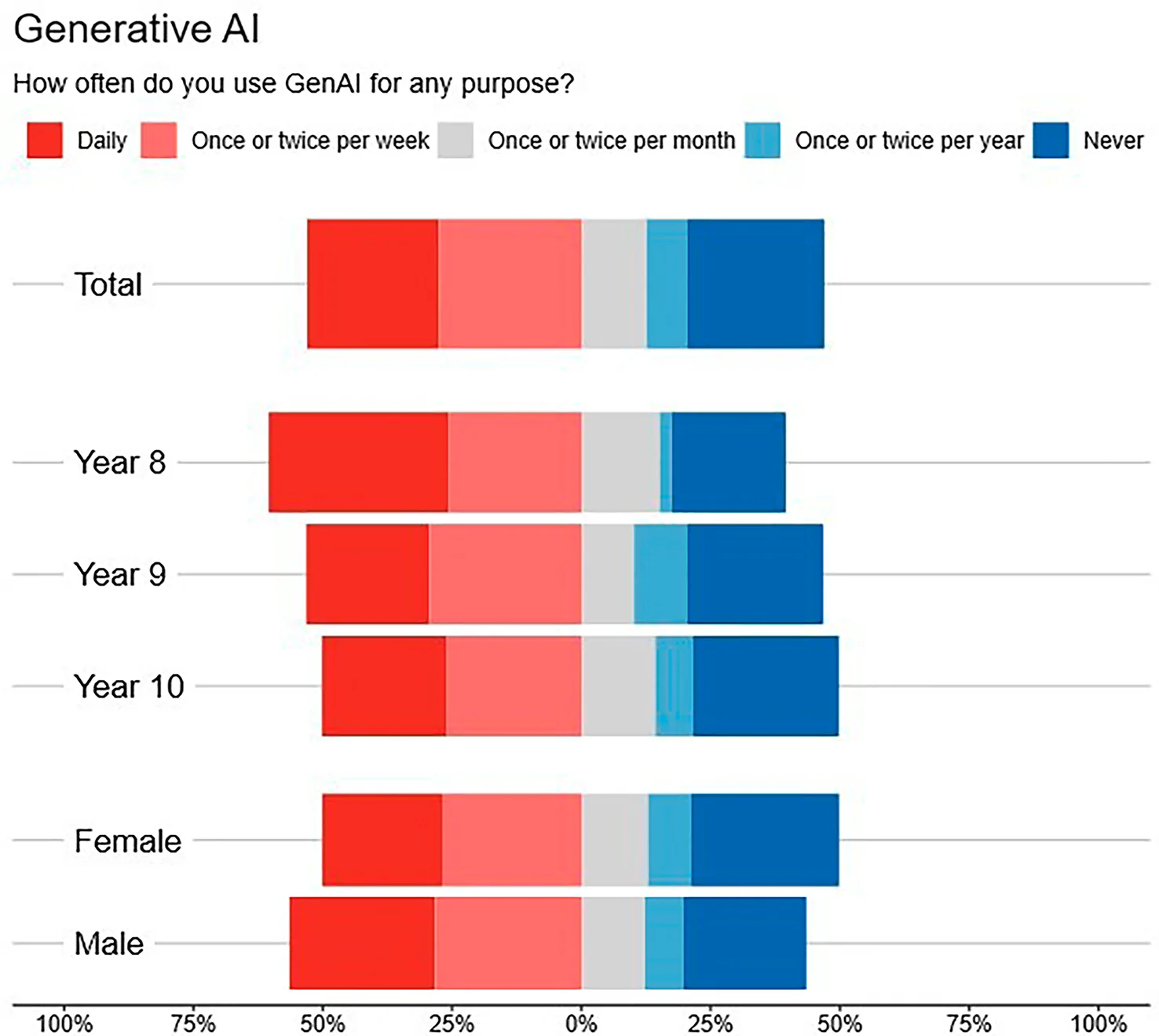
Use of GenAI for any purpose in Bradford, by year group and sex. Stacked bar charts show the percentage of respondents reporting Generative AI use for any purpose as ‘daily’, ‘once or twice per week’, ‘once or twice per month’, ‘once or twice per year’, or ‘never’. ‘Any purpose’ was derived from the highest frequency response option among the eight possible purposes (getting information; checking work; generating new ideas; creating images, sounds, or music; writing code; getting advice; schoolwork; and social reasons).

Daily use of GenAI was higher among males than females (28.3% vs 23.2%), with the largest sex differences observed for getting information (11.7% vs 6.1% daily) and checking work (13.6% vs 8.7% daily). Year 8 students reported higher daily use overall (34.9%) than Year 9 (23.8%) and Year 10 students (24.1%), with similar patterns observed for schoolwork (20.7%, 13.1%, and 11.9% respectively) and checking work (16.0%, 10.7%, and 9.4% respectively). Use for coding declined markedly across year groups, from 3.8% daily in Year 8 to 1.3% in Year 10.

Year 8 students reported higher daily use overall (34.9%) than Year 9 (23.8%) and Year 10 (24.1%), with the difference most evident for schoolwork (20.7%, 13.1%, 11.9%), checking work (16.0%, 10.7%, 9.4%), and creating images, sounds, or music (8.8%, 4.0%, 2.9%). Getting information and generating new ideas followed similar patterns, declining from Year 8 to Year 10 (9.6%, 9.5%, 7.5% and 10.3%, 8.8%, 7.2% respectively), though the decline was more gradual. Getting advice was the exception, with Year 9 students reporting the highest daily use (5.7%) compared to Year 8 (4.9%) and Year 10 (2.5%). This was also the only category females (4.6%) reported higher daily use than males (3.9%). Coding and social use were consistently low across all three year groups, with over three quarters of students in each year reporting never using GenAI for this purpose.

Regarding trust in GenAI outputs, 39.4% of participants reported trusting the answers provided ‘not at all’, and a further 13.7% reported ‘not really’ ( Table 3, Figure 2). Only 4.1% reported trusting AI outputs ‘entirely’. Males were more likely to report some level of trust (30.9% vs 18.9% among females) and less likely to report no trust at all (34.0% vs 44.0%). Males also reported more active verification – checking the answer it provides ‘almost always’ (11.7% vs 6.7% of females), while females were more likely to never verify (53.2% vs 40.7% of males; Table 3, Figure 3). With respect to age, Year 8 students were the most likely to report no trust at all (47.5%), compared to Year 9 (35.5%) and Year 10 (40.9%). Never verifying was the modal response across all three year groups (53.2%, 45.5%, and 47.8% respectively).

**Table 3.** Trust in and verification of GenAI outputs. When I use AI, I …

		Trust the answers it provides					Verify the answers it provides				
		Entirely	Somewhat	Undecided	Not really	Not at all	Almost always	Sometimes	Every once in a while	Rarely	Never
**Total**		**4.1**	**20.2**	**22.6**	**13.7**	**39.4**	**9.0**	**17.7**	**13.3**	**12.4**	**47.5**
Year Group	8	7.5	21.3	12.5	11.3	47.5	7.6	19.0	11.4	8.9	53.2
	9	4.4	21.8	25.8	12.5	35.5	9.0	18.4	16.8	10.2	45.5
	10	2.6	18.3	22.6	15.7	40.9	9.4	16.5	10.3	16.1	47.8
Sex	Female	4.2	14.7	21.2	16.0	44.0	6.7	14.4	13.7	12	53.2
	Male	3.9	27.0	24.2	10.9	34.0	11.7	21.8	12.9	12.9	40.7

**Figure 2.**
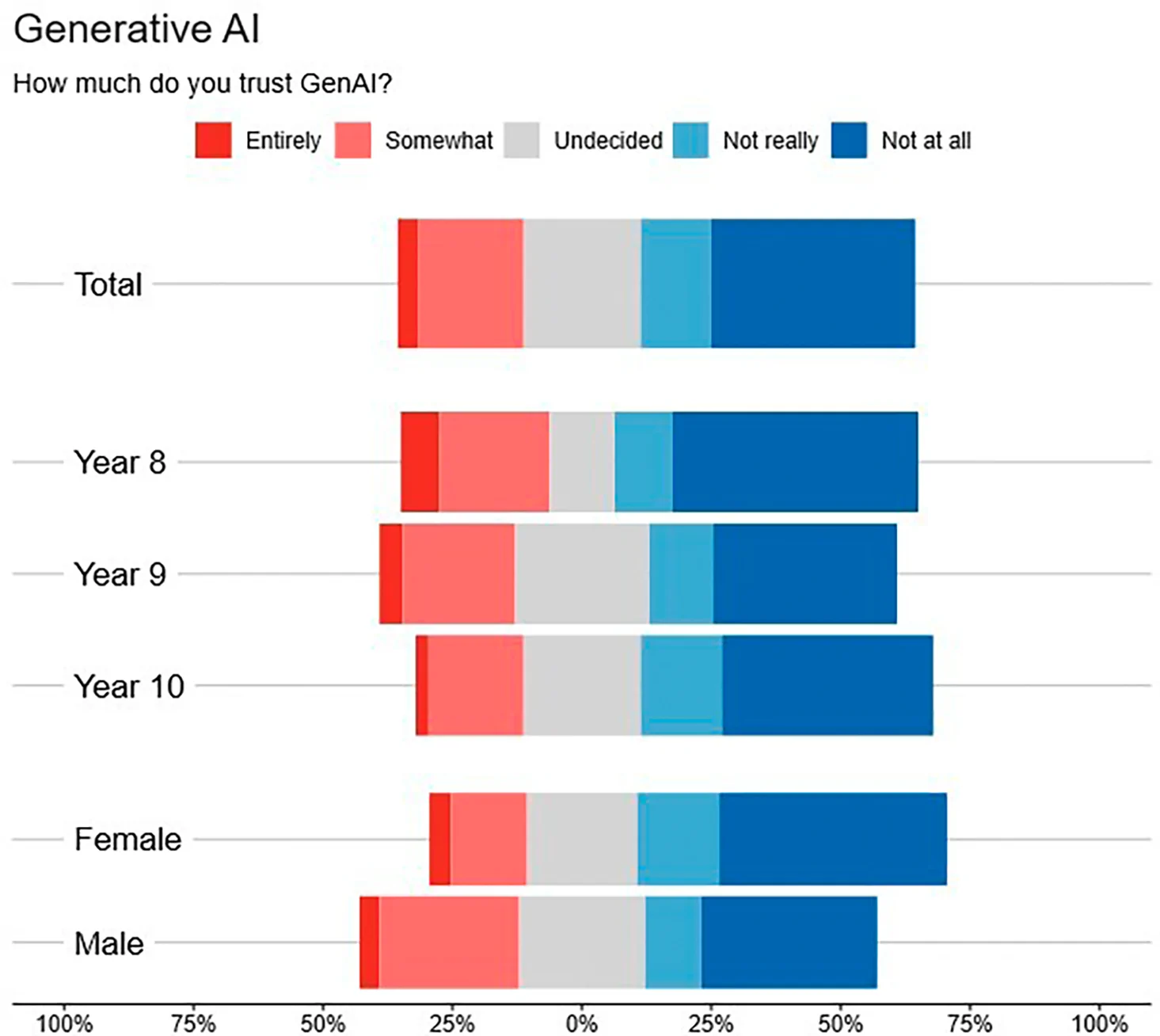
Perceived trust of GenAI in Bradford, by year group and sex. Stacked bar charts show the percentage of respondents reporting they trust GenAI ‘entirely’, ‘somewhat’, ‘not really’, ‘not at all’, or ‘undecided’.

**Figure 3.**
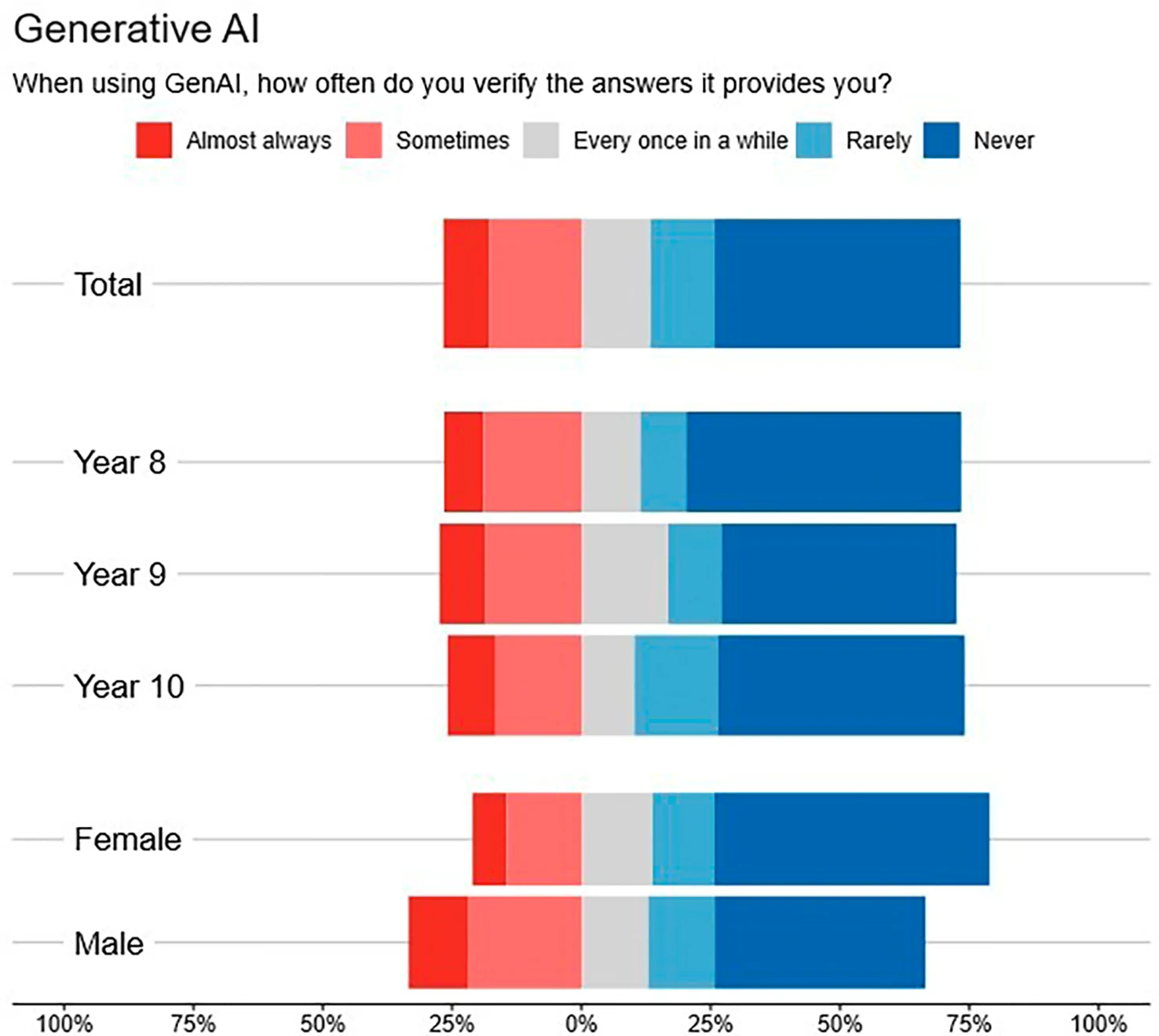
Frequency of verifying answers provided by GenAI in Bradford, by year group and sex. Stacked bar charts show the percentage of respondents reporting they verify the answers GenAI provides them ‘almost always’, ‘sometimes’, ‘every once in a while’, ‘rarely’, or ‘never’.

Perceived usefulness of GenAI for school subjects was generally low ( Table 4, Figures 4- 7). Across all four subject types, the modal response was ‘not at all useful’, with creative subjects rated least useful overall (41.1%) and problem-solving subjects (e.g., maths) receiving the highest proportion of ‘very much useful’ ratings (21.1%). Year 8 students rated GenAI as more useful than older year groups across all subjects, most notably for writing (23.5% rating 6 vs 8.9% in Year 10) and creative subjects (20.8% vs 7.8%). Males rated GenAI as more useful than females across all subjects, with the largest differences for creative subjects (15.0% vs 9.3%).

**Table 4.** Perceived usefulness of GenAI use across school subjects. I find GenAI useful for …

		Writing subjects (e.g., English, languages)						Problem-solving subjects (e.g., mathematics)					
		1 – Not at all	2	3	4	5	6 – Very much	1 – Not at all	2	3	4	5	6 – Very much
**Total**		**29.7**	**7.2**	**23.1**	**16.7**	**9.5**	**13.8**	**26.2**	**9.1**	**14.9**	**15.3**	**13.5**	**21.1**
Year Group	8	25.9	8.6	19.8	14.8	7.4	23.5	26.9	7.7	10.3	12.8	16.7	25.6
	9	23.1	5.8	26.9	20.2	8.7	15.3	21.9	8.9	17.0	18.2	13.0	21.1
	10	37.9	8.1	20.4	13.6	11.1	8.9	30.7	9.8	14.2	12.9	12.9	19.6
Sex	Female	31.7	6.6	22.8	17.2	7.9	13.9	28.0	8.7	14.0	17.7	12.0	19.7
	Male	27.5	7.8	23.5	16.1	11.4	13.7	24.0	9.6	16.0	12.4	15.2	22.8

		Knowledge-based subjects (e.g., geography, science)						Creative subjects (e.g., art)					
		1 – Not at all	2	3	4	5	6 – Very much	1 – Not at all	2	3	4	5	6 – Very much
**Total**		**29.8**	**6.5**	**18.1**	**19.2**	**10.2**	**16.2**	**41.1**	**8.8**	**16.4**	**13.7**	**8.1**	**11.9**
Year Group	8	28.2	6.4	19.2	10.3	9.0	26.9	28.6	10.4	18.2	15.6	6.5	20.8
	9	25.5	6.9	19.0	23.9	10.5	14.2	35.0	9.8	17.1	17.9	7.3	13.0
	10	34.9	6.0	16.8	17.2	10.3	14.7	51.7	7.3	15.1	8.6	9.5	7.8
Sex	Female	32.7	8.3	16.7	20.3	8.7	13.3	46.8	8.6	12.3	15.0	8.0	9.3
	Male	26.5	4.3	19.8	17.9	12.1	19.5	34.3	9.1	21.3	12.2	8.3	15.0

**Figure 4.**
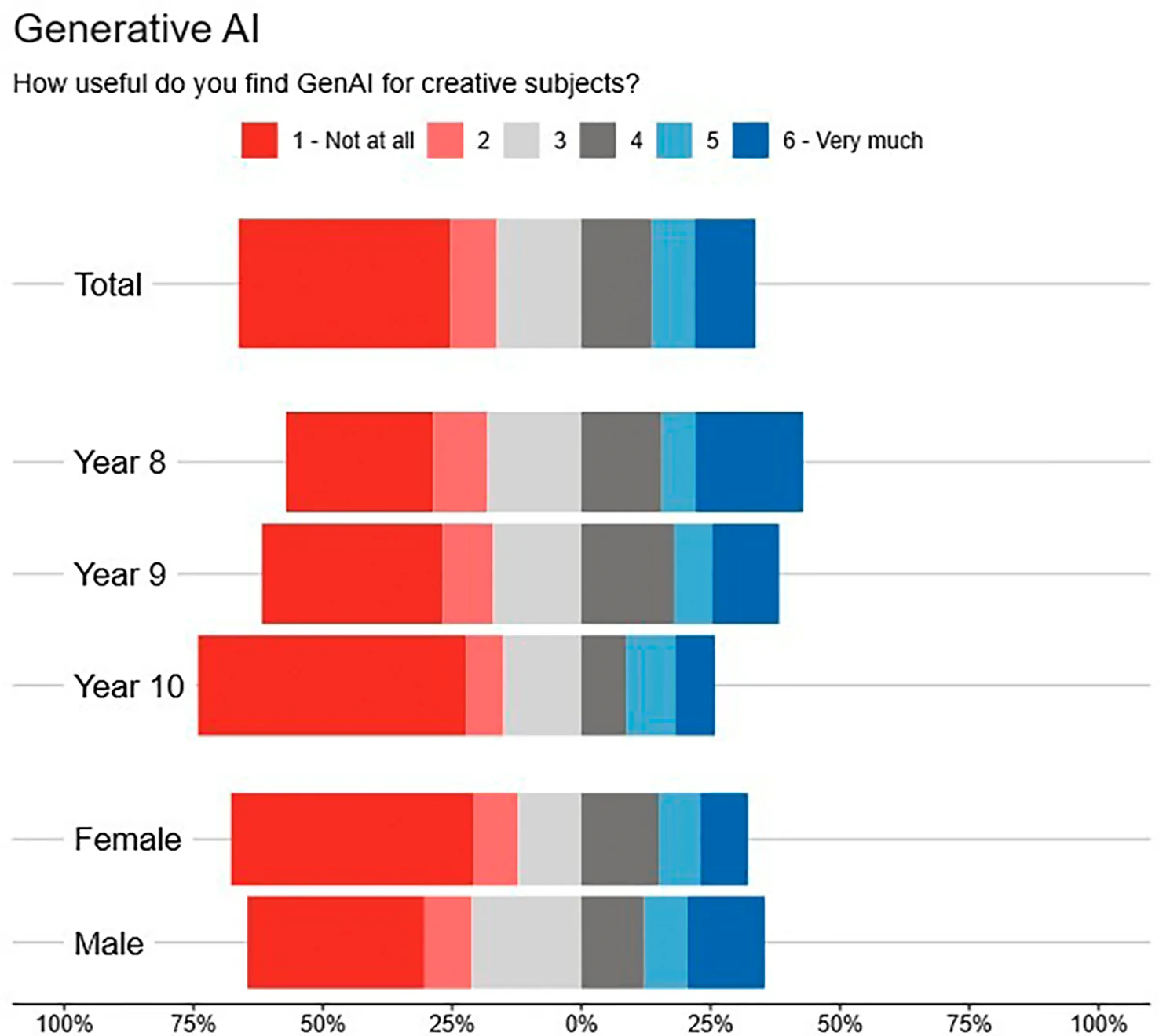
Perceived usefulness of GenAI for writing subjects in Bradford, by year group and sex. Stacked bar charts show the percentage of respondents rating the usefulness of GenAI for writing subjects from ‘1 – Not at all’ to ‘6 – Very much’. Examples of writing subjects may include English or languages.

**Figure 5.**
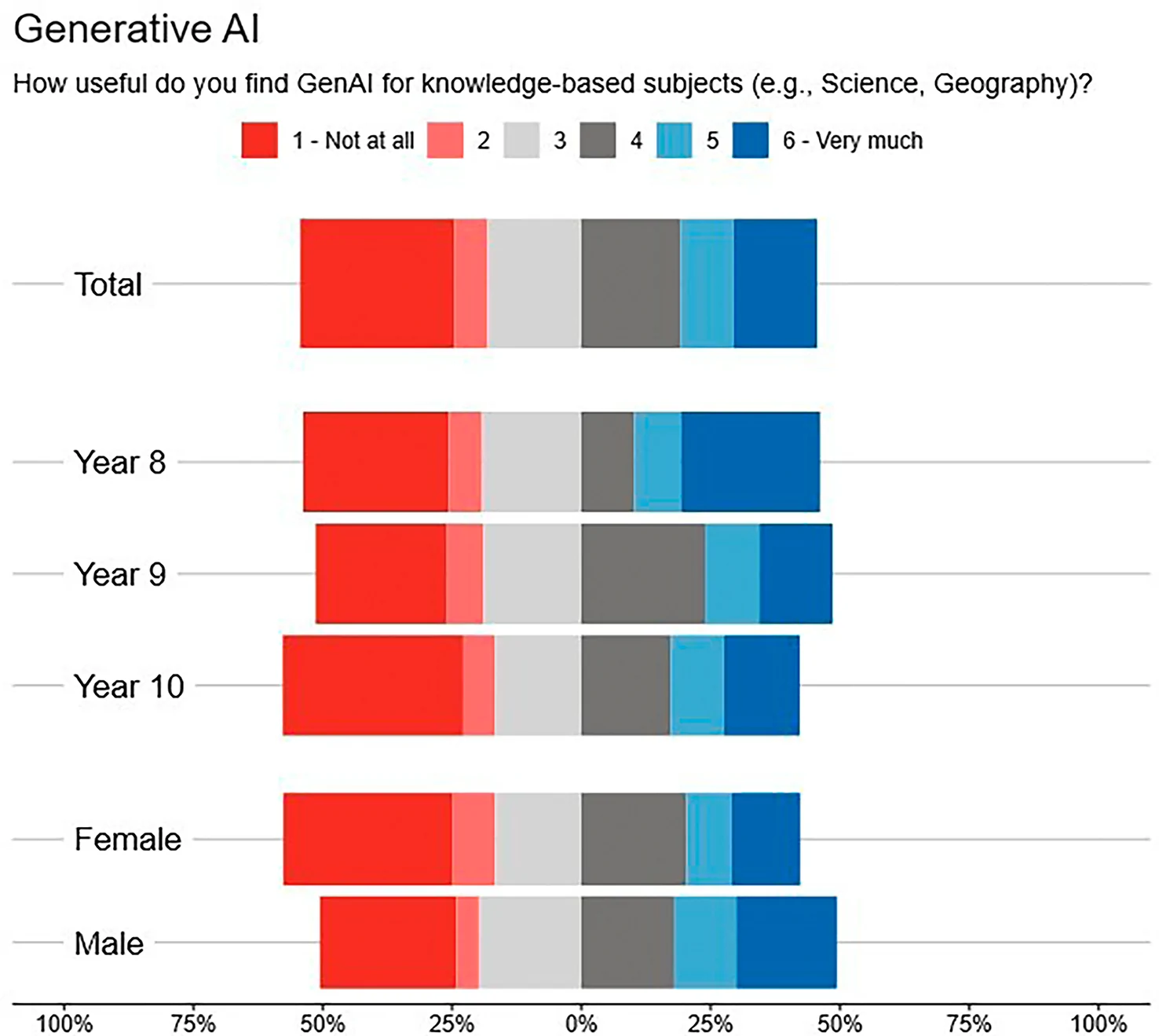
Perceived usefulness of GenAI for problem-solving subjects in Bradford, by year group and sex. Stacked bar charts show the percentage of respondents rating the usefulness of GenAI for writing subjects from ‘1 – Not at all’ to ‘6 – Very much’. An example of a problem-solving subject may include maths.

**Figure 6.**
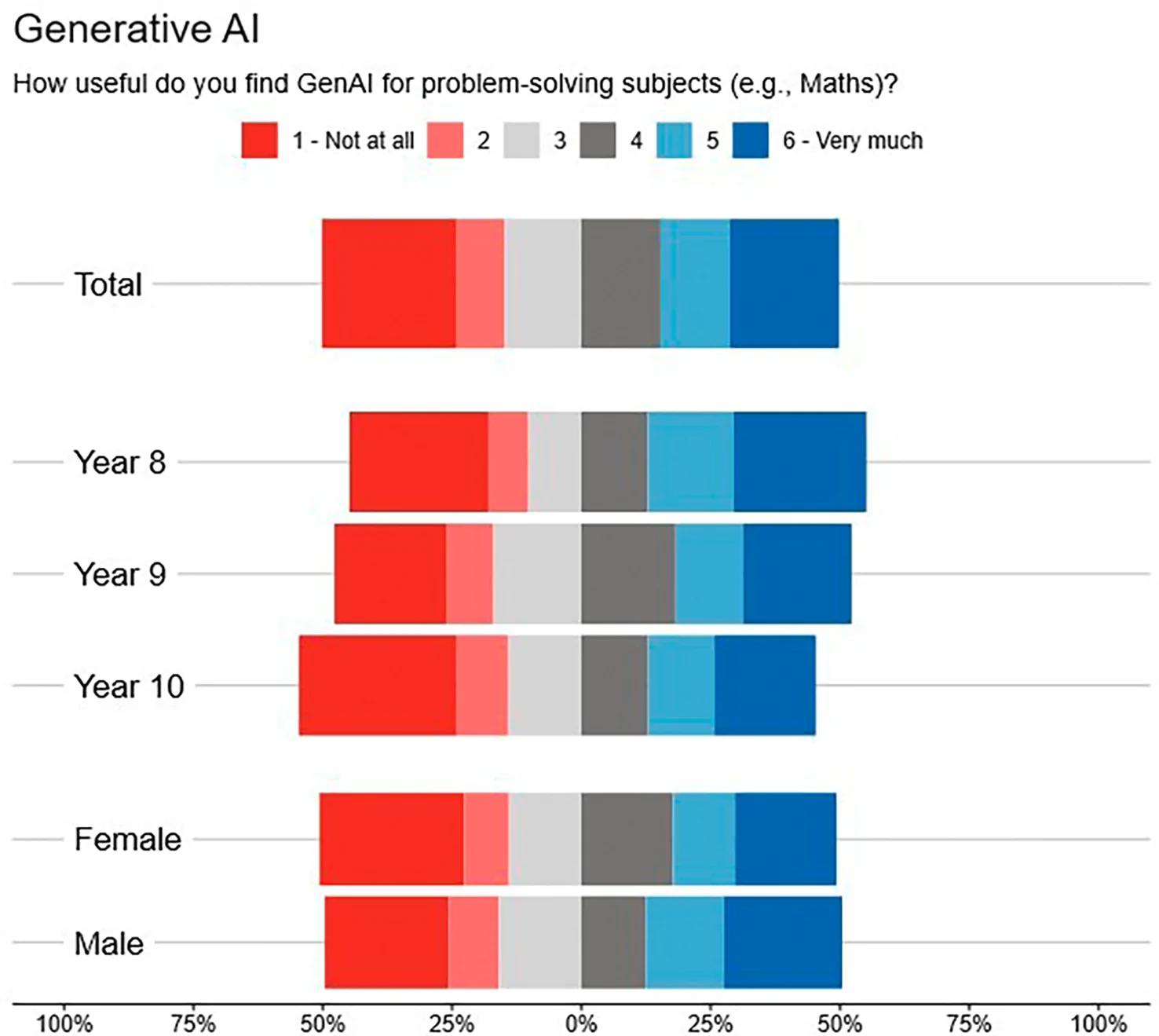
Perceived usefulness of GenAI for knowledge-based subjects in Bradford, by year group and sex. Stacked bar charts show the percentage of respondents rating the usefulness of GenAI for knowledge-based subjects from ‘1 – Not at all’ to ‘6 – Very much’. Examples of knowledge-based subjects may include geography or science.

**Figure 7.**
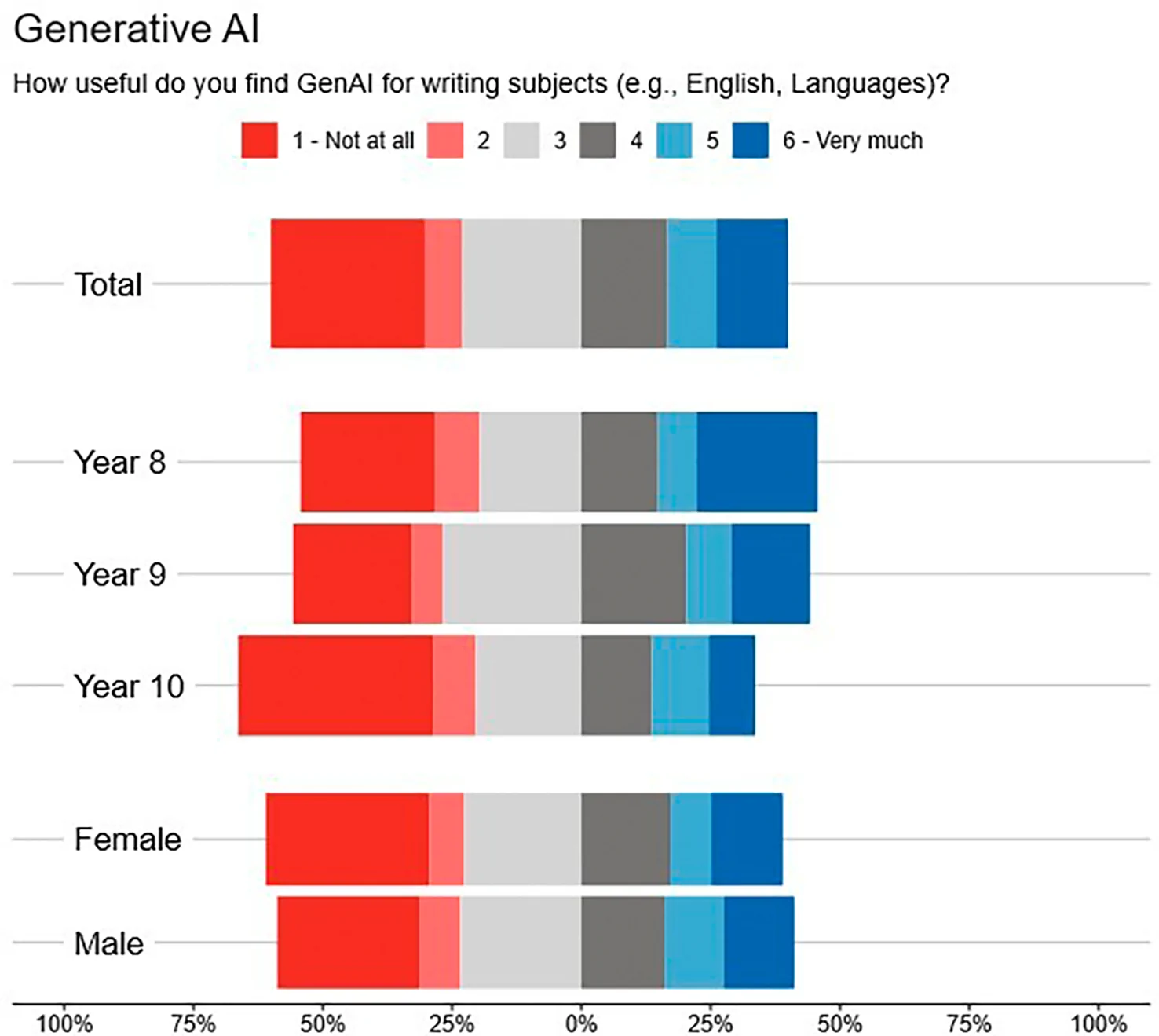
Perceived usefulness of GenAI for creative subjects in Bradford, by year group and sex. Stacked bar charts show the percentage of respondents rating the usefulness of GenAI for creative subjects from ‘1 – Not at all’ to ‘6 – Very much’. Examples of creative subjects may include art.

## Discussion

This study provides an early descriptive account of GenAI use among adolescents in Bradford, UK. The majority of participants had used GenAI at least once, with schoolwork the dominant purpose. Trust in AI outputs was low across the sample, yet most participants never regularly verified them. Perceived usefulness for school subjects was generally low, with creative subjects rated least useful across all groups.

### Prevalence and purposes of use in context

The prevalence of GenAI use observed in this sample – around three-quarters of students reporting at least occasional use – is broadly consistent with national survey data for UK teenagers, though direct comparisons are limited by differences in sampling frames, age ranges, and question wording. ^15^ The concentration of use around academic purposes mirrors national patterns, with homework help and information-seeking among the most commonly reported uses of GenAI among young people in the UK. ^15^ The low prevalence of social use in this sample is notable given concerns raised elsewhere about emotional reliance on AI chatbots, ^3^ ^,^ ^16^ ^,^ ^17^ though whether this reflects limited platform access, cultural context, or genuine disinterest in social GenAI use cannot be determined from these descriptive data.

### Year group differences

Higher GenAI use among Year 8 students, particularly for academic purposes, indicate that GenAI adoption does not simply rise with age. National data suggest that copying GenAI outputs without modification is more common among younger adolescents. ^15^ Year 10 students approaching formal GCSE assessment may also be more aware of potential sanctions for GenAI use in assessed work, or more cautious about self-reporting use in a school-administered survey context. The lower perceived usefulness among Year 10 students may reflect the fact that they were older when GenAI tools became widely accessible, with more developed academic skills and established study habits at the point of adoption.

### Sex differences

Males reported higher GenAI use, greater trust in GenAI outputs, and more active verification than females across nearly all measures. These patterns are consistent with broader evidence that male students report higher GenAI use and confidence across academic contexts, while female students approach these tools more cautiously and express stronger concerns about reliability and accuracy. ^18^ ^,^ ^19^ What is notable is that both sexes showed similarly low rates of active verification despite differing levels of trust, suggesting that low verification behaviour may reflect a broader gap in GenAI literacy provision.

### Perceived usefulness

Perceived usefulness of GenAI for school subjects was generally low. Creative subjects were rated least useful overall while problem-solving subjects were rated most useful. Perceived usefulness declined between Year 8 and Year 10 across all subjects, consistent with the broader pattern of lower engagement among older students, though whether lower perceived usefulness among older students reflects reduced use, greater familiarity with GenAI limitations, or other factors cannot be determined from these descriptive cross-sectional data.

### Strengths and limitations

Key strengths of this study include the use of school-administered survey data linked to administrative records which enable robust demographic characterisation, purpose-built GenAI questions administered within an established cohort infrastructure, and a focus on socioeconomic and ethnic demographics underrepresented in existing research on adolescent GenAI use. The Born in Bradford Age of Wonder study is well placed to examine these questions longitudinally as GenAI use among young people continues to evolve.

Several limitations should be acknowledged. All GenAI use data were self-reported and subject to recall and social desirability bias. Due to coming from a pilot of the questionnaire, the sample is small and drawn from three schools, limiting generalisability. These were inner-city schools with a substantial overrepresentation of South Asian/South Asian British, Pakistani-heritage students and those living in the most deprived IMD quintile. Therefore, findings should not be taken as representative of Bradford as a whole or of England more broadly. Further, analyses are descriptive and cross-sectional, precluding causal inference or estimation of associations between GenAI use and health or educational outcomes.

Future analyses using the full 2025/26 cohort will enable more precise estimates, broader demographic coverage, and examination of associations between GenAI use and mental health and wellbeing outcomes.

## Ethics statement

The Age of Wonder study is approved by the Bradford Leeds NHS Research Ethics Committee [Ref: 21/YH/0261, date: 22.12.21]. The consent process is described in the study protocol. ^20^ In brief, parents received information sheets about the survey and provided opt-out consent, while participants provided assent.

## Data Availability

Raw data from Born in Bradford cannot be made publicly available, following our ethical approvals and commitment to protect participants’ confidential information. Researchers can apply to access individual-level data, with information about this process available at https://borninbradford.nhs.uk/our-data/how-to-access-data/. Open Science Framework – AI use and perceptions (2024–25). https://doi.org/10.17605/OSF.IO/SQMDR ^14^
• Supplementary figures and tables • R code for producing tables and plots • BiB: Age of Wonder Young People’s Survey for academic year 2024/25 Data are available under the terms of the Creative Commons Zero “No rights reserved” data waiver (CC0 1.0 Public domain dedication).
