# Supplementary material for: Use and perceptions of Artificial Intelligence among adolescents in Bradford, England: Evidence from Born in Bradford: Age of Wonder
Source: Wellcome Open Res. 2026 Aug 17;11:525. [Version 1] doi: 10.12688/wellcomeopenres.27280.1 (PMC13728437; doi:10.12688/wellcomeopenres.27280.1)
Supplement: Peer review history

# Reviewer report: Reviewer response for version 1

**DOI:** 10.21956/wellcomeopenres.30024.r175502

**Published:** 2026-11-02

**Authors:** Oluwaseun Emmanuel Omopo

**Affiliations:** 1 University of Johannesburg, Auckland Park, Gauteng, South Africa

Thank you for the opportunity to review this paper, which explores the use of and attitudes towards GenAI among adolescents living in Bradford, England. The study makes a valuable addition to the literature, particularly in this population group, by exploring an under-represented sample of children from socioeconomically deprived, ethnically diverse backgrounds. The authors clearly state the rationale for the dataset they have created and describe the use of an existing population cohort well. The results are important for understanding how often and for what purposes GenAI is used, as well as user trust, behaviour and utilitarian value for learning. However, some clarifications are needed around the methodology, in particular, participant selection from the three schools, a more detailed explanation of missing data, and the derivation of the new measure of GenAI use. Although differences are found across sex and school year groups, all differences presented are purely descriptive, and as such, the paper should not suggest that these are statistically significant differences. Lastly, low trust and verification should be interpreted with caution as a possible gap in AI literacy, as this construct was not directly assessed.

 In addition, there is room for improvement in the reporting of the findings for clarity and consistency. For example, the "nine purposes" of GenAI use identified in the manuscript need to be reviewed and revised to reflect the eight purposes reported under the Methods section. Also, the captions and headings for Figures 4–7 need to be revised to match each other, and the results need to be expressed more clearly. The supplementary datasets need to be reported in a little more detail regarding whether and where the variable descriptions, analytical procedures and datasets can be found. The limited reference to the literature and lack of a well-developed discussion of implications for teachers, education practitioners and educators are also drawbacks.

 Overall, this is a worthwhile study that is appropriate for indexing as a Data Note, provided that it is acceptable to index with methodological, reporting and interpretative caveats. Approval is recommended, subject to revisions.

Are sufficient details of methods and materials provided to allow replication by others?

Yes

Is the rationale for creating the dataset(s) clearly described?

Yes

Are the datasets clearly presented in a useable and accessible format?

Yes

Are the protocols appropriate and is the work technically sound?

Yes

Reviewer Expertise:

child and adolescent psychology, clinical psychology, educational psychology

I confirm that I have read this submission and believe that I have an appropriate level of expertise to confirm that it is of an acceptable scientific standard.

# Reviewer report: Reviewer response for version 1

**DOI:** 10.21956/wellcomeopenres.30024.r168785

**Published:** 2026-10-09

**Authors:** Eugene Lee Davids

**Affiliations:** 1 University of Pretoria, Pretoria, South Africa

Thank you for the opportunity to review “Use and perceptions of Artificial Intelligence among adolescents in Bradford, England: Evidence from Born in Bradford: Age of Wonder”. The paper sets out to explore the use of and perceptions of Generative AI among a group of adolescents who have largely been excluded from existing research. At a time when the use of Generative AI is becoming more commonplace, this study is both timely and needed.

 After reviewing this very interesting paper, I provide the following suggestions to further strengthen the paper in its current form:

  

- For the differences presented, were they statistically different? It might be helpful for the reader to determine whether the differences across the variables and groups (grade/year and sex) are statistically significant or not.
- Another suggestion would be to determine whether there are statistically significant differences between the Bradford data among the study participants, as well as that within the overall Bradford area and nationally, as outlined in Table 1 of the sample characteristics.
- The discussion would have benefited from more engagement with available literature. It would have also been very helpful to reflect on the usefulness of the findings for (i) teachers and school practitioners, (ii) school support initiatives, and (iii) generally the usefulness of the findings for policy and practice.

Are sufficient details of methods and materials provided to allow replication by others?

Yes

Is the rationale for creating the dataset(s) clearly described?

Yes

Are the datasets clearly presented in a useable and accessible format?

Yes

Are the protocols appropriate and is the work technically sound?

Yes

Reviewer Expertise:

Adolescence; Adolescent Health; Adolescent Health Decision-Making

I confirm that I have read this submission and believe that I have an appropriate level of expertise to confirm that it is of an acceptable scientific standard.
